# Concurrent Chorioptic Mange and Dermatophytosis in Dairy Goats: A Case Report

**DOI:** 10.3390/vetsci9120677

**Published:** 2022-12-06

**Authors:** Luisa Rambozzi, Pier Giuseppe Meneguz, Anna Rita Molinar Min, Mario Pasquetti, Andrea Peano

**Affiliations:** Department of Veterinary Sciences, University of Turin, L. Go P. Braccini 2, Grugliasco, 10095 Turin, Italy

**Keywords:** goat, chorioptic mange, *Trichophyton verrucosum*

## Abstract

**Simple Summary:**

*Chorioptes* mites are particularly common in goats, with infestations usually subclinical and often asymptomatic. In the present case, many animals had a severe clinical presentation, possibly due to the associated dermatophyte infection (*Trichophyton verrucosum*), since a concurrent or underlying disease may exacerbate clinical response. Goats were treated topically with pour-on Eprinomectin (1 mg/kg), while an enilconazole solution was used for environmental disinfection against dermatophyte spores.

**Abstract:**

A concurrent chorioptic mange and dermatophytosis outbreak occurred in a goat flock in northwestern Italy. Sanitation of the flock was obtained following pour-on eprinomectin application at a dose of 1 mg/kg; enilconazole was used for environmental disinfection against dermatophyte spores.

## 1. Introduction

The surface-living mite, *Chorioptes bovis*, causes chorioptic mange; the infestation affects livestock species such as cattle, horses, sheep, and alpacas and is also a common problem in goat farming worldwide [1,2,3,4,5,6]. The mites are found principally on the feet of animals, where they cause crust formation (the so-called “foot mange”) [4]. Other clinical presentations are possible, such as small crusts hidden under the hair coat. As the mite feeds on skin debris, there is rarely severe skin damage, resulting in subclinical infection, especially in goats. *Chorioptes* infection is often observed on farms with poor management and nutrition [4].

Dermatophytosis, or “ringworm”, is a fungal skin infection representing a significant problem in veterinary medicine with substantial consequences on public health [7]. Indeed, all animal-associated dermatophytes can infect humans [8,9,10]. Dermatophytes also include anthropophilic species, strictly human pathogens, and geophilic species. These latter reside in the soil, where they degrade keratinous materials spread in the environment by mammals and birds [7].

Each zoophilic dermatophyte recognises a few animal species as primary hosts. The most important species are *Microsporum canis*, associated with cats, *Trichophyton erinacei* with hedgehogs, *Trichophyton mentagrophytes* with rabbits, and *Trichophyton benhamiae* with guinea pigs [7,11,12]. *Trichophyton verrucosum* is largely diffused as the causative agent of cattle ringworm [7,13,14], with enzootic situations commonly occurring in herds worldwide [8,15,16,17]. Infection occurs mainly through direct transmission from animal to animal; therefore, a high prevalence can be reached in overcrowded stables where the fungus can spread quickly among subjects confined to small areas. The environmental fungal propagules also represent a significant infection source [18].

Ringworm in cattle may negatively impact milk and meat production and lead to impairments in the hide industries, as lesion scars can persist on leather following tawing and tanning [7]. Moreover, *T. verrucosum* has a high zoonotic potential [8,15,19,20]. Farmers, their families, veterinaries and technicians involved in animal management are at higher risk of infection. Lesions are usually highly inflammatory due to an allergic reaction elicited by the fungus in the stratum corneum. This disease is commonly called “barn itch” [20].

Dermatophytosis is considered a more sporadic disease in goats, with fewer cases reported in the literature. *T. verrucosum* and *T. mentagrophytes* are the most frequently reported species [14,21,22,23,24,25,26]. 

This article describes an episode of an unusually concurrent infection in dairy goats caused by *T.verrucosum* and *C. bovis.*

## 2. Materials and Methods

### 2.1. History and Signalment

**Figure 1:**
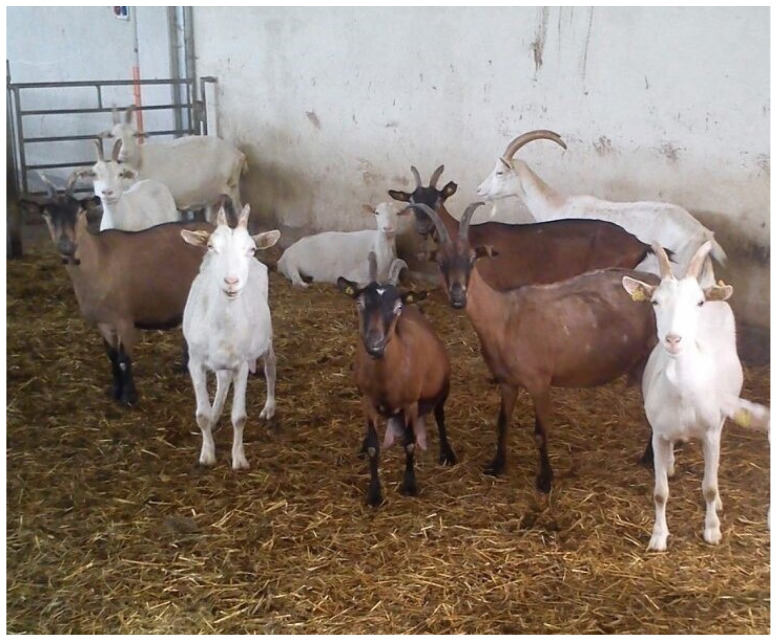
Some of the goats housed on the farm where dermatological problems were referred.

The outbreak involved a farm of 152 dairy goats of the Alpine (*n* = 83), and Saanen (*n* = 79) breeds in North-Western Italy. The farmer reported that skin lesions, mainly alopecia and crusts, had been present in many Alpine goats for about two months, while Saanen goats did not seem affected. The animals were housed without breed partition, and the management was the same for both breeds (Figure 1).

None of the animals exhibited itching or other symptoms; appetite and milk production, recorded by automated milk sensors, were not affected. The vaccination and deworming status of the animals (fenbendazole administration in the dry period) were up to date.

### 2.2. Clinical Examination and Samplings

The individual clinical examination was performed on all animals. Samples, including skin scrapings, scab materials and hair samples, were taken from 10 animals with skin lesions (all Alpine) and 10 randomly selected asymptomatic animals (all Saanen).

### 2.3. Laboratory Procedures

Samples were digested in 10% sodium hydroxide (NaOH) for three hours at room temperature and centrifuged at 3000 rpm for 10 min. After the supernatant was removed, the remaining material was transferred to slides and observed for mites and fungal elements. 

Fungal cultures were performed on a commercial medium for dermatophyte isolation (Mycobios Selective Agar, Biolife, Milan, Italy) and Sabouraud Dextrose agar enriched with thiamine and inositol. The latter medium was employed to facilitate the growth of *T. verrucosum*. For the same reason, the plates were incubated at 37 °C [27]. The culture media included antibiotics and cycloheximide to limit the growth of contaminant organisms. The fungal identification was obtained by analysing the colonies’ gross and microscopic morphology compared with standard descriptions [28].

## 3. Results

**Figure 2:**
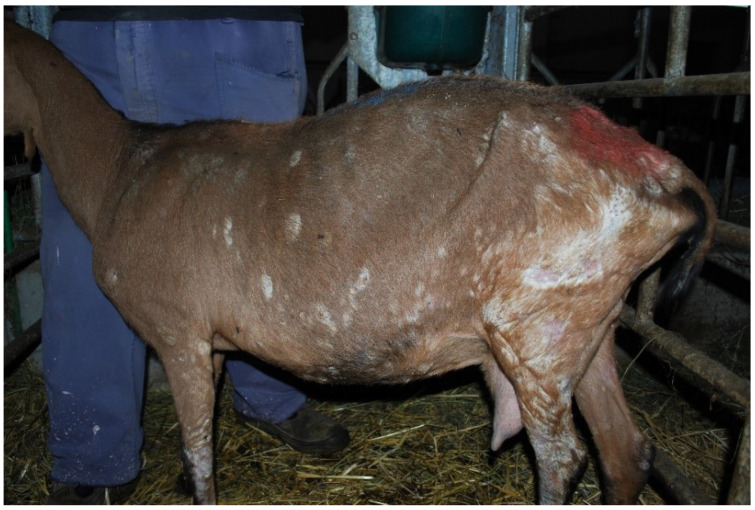
Extensive exfoliative and crusty dermatitis in a goat infected by *C. bovis* and *T. verrucosum*.

The clinical examination revealed 45 symptomatic animals belonging to the Alpine breed (54.2%). Lesions included multifocal alopecia on the chest and croup, crusts, thickening, and wrinkling on legs, axillae, inguinal and perineal region (Figure 2). 

None of the Saanen goats exhibited symptoms.

All ten skin samples from symptomatic animals resulted positive for *C. bovis*. 

**Figure 3:**
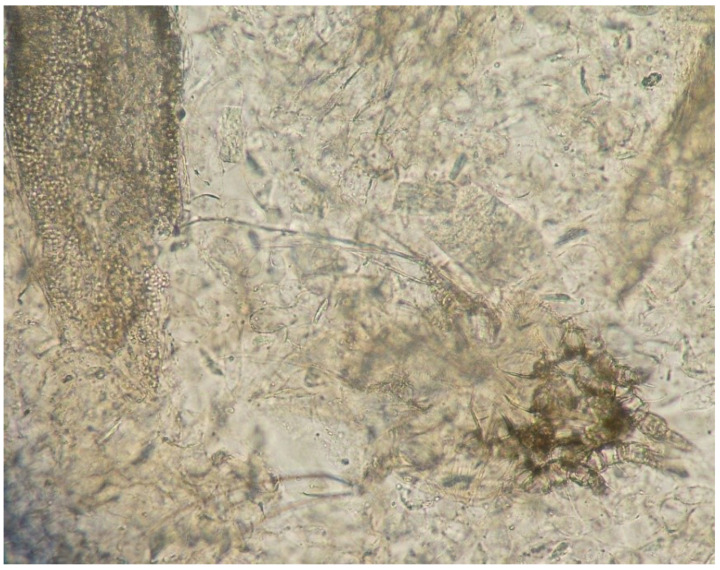
Direct examination of a skin scraping: hair invaded by fungal structures (arthroconidia) (left) and *C. bovis* mite (right) (10× magnification).

**Figure 4:**
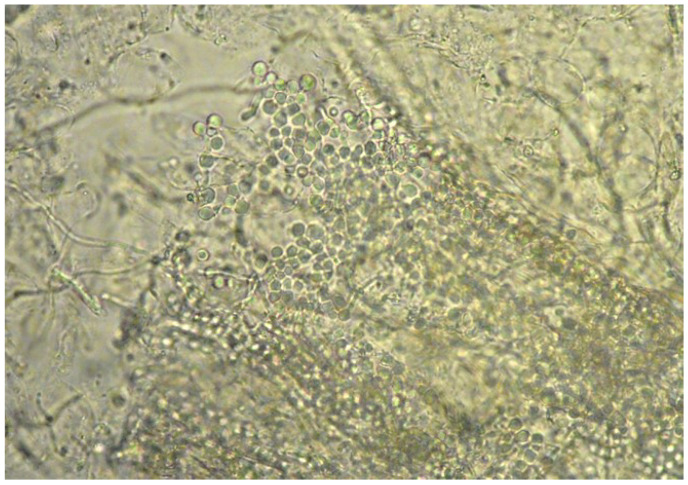
Direct examination of a skin scraping: hair invaded by fungal structures (arthroconìdia) (40× magnification).

Moreover, in eight of these samples, hairs were invaded by fungal structures with morphology suggestive of *T. verrucosum* (hyphae and chains of large ectothrix arthroconidia) (Figure 3 and Figure 4). 

Cultures yielded flat, white/cream colour, with glabrous texture colonies. The growth was slow, with colonies evident only after prolonged incubation (around two weeks). The isolation was difficult due to a large number of contaminant mould colonies. Irregular hyphae were observed to have terminal and intercalary chlamydospores. The chlamydospores were arranged in characteristic long chains. Based on the results of direct examination and cultures, the fungus was identified as *T. verrucosum.*

No mites or dermatophytes were detected in asymptomatic goats.

All goats were treated topically with pour-on Eprinomectin (EPM) (Eprinex^®^ Multi Pour-On- Boehringer Ingelheim Animal Health) at 1 mg/kg. Enilconazole solution (Clinafarm Spray^®^, Ely Lilli Italia S.p.A.) was used for environmental disinfection against dermatophyte conìdia.

The response to treatment was evaluated clinically, and skin scraping and fungal culture were performed weekly for up to 2 consecutive negative scrapings.

After two months, all skin samples gave negative results, and all the goats were fully clinically recovered. 

## 4. Discussion

*Chorioptes* mites are particularly common in goats [2,3], with infestations usually subclinical [4] and often asymptomatic [29]. In the present case, many animals had a severe clinical presentation, possibly due to the associated dermatophyte infection, since clinical response may be exacerbated by a concurrent or underlying disease [5,30]. Lesions on the animals (alopecia with scaling and crusts) were similar to those described elsewhere in goats with dermatophytosis [14,23].

All positive goats were of the Alpine breed; this may be related to the coat’s colour (white in the Saanen breed; brown in the Alpine breed). Indeed, there is evidence that hair colour influences resistance to ectoparasites. Darker individuals appear more attractive to flies [31,32,33], ticks [3,33,34,35] and mites [36] than their paler counterparts.

Concerning ringworm in livestock, cattle are generally considered the more at risk of problems [7], especially in intensive and semi-intensive farming systems. In these situations, infection rates often reach very high values due to a combination of factors, such as the presence of young animals without acquired immunity, overcrowding of animals, and increased environmental humidity and temperature [8,16]. The most frequent dermatophyte species reported in the literature infecting bovines is *T. verrucosum* [7]. 

Few studies on goat ringworm are available. The prevalence and importance of this fungal infection in goat farming appear extremely variable from study to study. A retrospective study covering 20 years of medical records at the School of Veterinary Medicine, University of California, Davis, revealed a very low prevalence of animals with dermatophytosis out of a population of goats with skin problems. The only species isolated was *T. mentagrophytes* [23]. Similarly, in a Pakistan study, only 0.2% of goats proved to be infected (by *T. verrucosum*) [14]. Moreover, the infection frequency was significantly lower than in cattle [14], reinforcing the idea that cattle are the main reservoir of *T. verrucosum*. In a study performed in Nigeria, one goat out of 13 examined was positive for a dermatophyte species [24]. The fungus isolated was *Trichophyton schoenleinii*, an anthropophilic dermatophyte. The explanation provided by the authors for this finding was that humans and sampled animals shared the same environment. Therefore the goat may have acquired the infection following contact with spores derived from infected humans or a contaminated environment. Another study reporting a low prevalence of infection regards goats in India. Two animals out of 28 were positive for *T. mentagrophytes* and *Microsporum gypseum* (now called *Nannizia gypsea*, a geophilic species), respectively [25]. Curiously, two other surveys performed in the same countries mentioned above (Nigeria and India) reported a higher prevalence of infected animals. In Nigeria, Emenuga and Oyeka [26] found 11 (13.8%) and 16 (20%) goats out of 80 sampled positive for *T. verrucosum* and *T. mentagrophytes*, respectively. In India, Begum and Kumar [22] reported around half of the goats (52.2%) were positive for various dermatophyte species. *T. mentagrophytes* and *T. verrucosum* accounted for the majority of cases. 

Despite the zoonotic potential of *T. verrucosum*, human involvement apparently did not occur in the present case. It is not easy to find an explanation for this lack of contagion since it is known that the infection can easily spread from animals to humans [8,15,19]. Humans contract *T. verrucosum* by contact with infected animals, contaminated fomites, or soil. The disease often follows local trauma, employed by the fungus as an entry portal [20]. Generally, the condition is highly evident since it presents as a well-circumscribed erythematous and pruriginous plaque covered with vesicles and papules. Clinical findings also include constitutional symptoms, especially fever and regional lymphadenopathy [20].

Because dermatophytosis is infectious and contagious, rapid confirmation of the disease is necessary to limit its spread. Different diagnostic tests can be used for this purpose.

Dermoscopy is a noninvasive point-of-care diagnostic tool that allows for illuminated skin magnification. It is widely used in human medicine to diagnose causes of hair and follicular abnormalities, including dermatophytosis [37]. Concerning veterinary medicine, this tool’s use is limited to canine and feline dermatology [38].

Direct examination of skin samples is instead routinely employed [7,13]. It is a quick and inexpensive test which allows putting in evidence hyphae and conìdia invading hair and scales. When the sample includes abundant and thick material rich in keratin (scales, crusts), as in the present case, it is necessary to digest it in NaOH or potassium hydroxide (KOH) to allow the visualisation of the fungal elements [14]. This method could rapidly detect the organisms (*Chorioptes* and *Trichophyton*) responsible for the dermatological problems affecting our goats. Though the definitive fungal identification was achieved later by culture, the morphology of the fungal elements on direct microscopy was already highly suggestive of *T. verrucosum*. The large (about 10 µm size) arthroconìdia, in chains or groups, were pretty typical of *T. verrucosum* and allowed differentiation of this fungus from other dermatophytes affecting livestock, e.g., *T. mentagrophytes*.

Diagnosis based on direct examination has a very high sensitivity concerning dermatophytosis by *T. verrucosum* in animals [8]. Indeed, infected crusts and scales generally contain infected hair that can be easily identified, provided the sample is adequately cleared in a NaOH or KOH solution. 

Culture is a useful diagnostic test since it allows a definitive identification through morphological and molecular analyses [7,13]. In our case, the cultures took several days to be positive since *T. verrucosum* grows very slowly, even on enriched culture media [14]. The poor growth of *T. verrucosum* often represents a severe problem for its isolation and identification, mainly due to the rapid development of a variety of non-pathogenic moulds that contaminate the hair of animals [7].

More advanced tests for dermatophytosis diagnosis are based on detecting fungal DNA from hair and scales through various PCR techniques [39]. Though not yet primarily employed in routine practice, these methods have also been proven valid for diagnosing animal infections, including cattle ringworm due to *T. verrucosum* [40].

Concerning the treatment of goats, we preferred to encompass the asymptomatic ones since we could not completely rule out the possibility of animals with subclinical lesions. Leaving untreated animals could have led to a disease spread, nullifying the efficacy of the EPM treatment. Moreover, EPM administration (with endectocide activity and with zero milk-withdrawal period) substituted the antihelmintic treatment (fenbendazole) routinely applied in the dry period.

## Data Availability

Not applicable.
